# New tris- and pentakis-fused donors containing extended tetrathiafulvalenes: New positive electrode materials for rechargeable batteries

**DOI:** 10.3762/bjoc.11.128

**Published:** 2015-07-08

**Authors:** Shintaro Iwamoto, Yuu Inatomi, Daisuke Ogi, Satoshi Shibayama, Yukiko Murakami, Minami Kato, Kazuyuki Takahashi, Kazuyoshi Tanaka, Nobuhiko Hojo, Yohji Misaki

**Affiliations:** 1 Department of Applied Chemistry, Graduate School of Science and Engineering, Ehime University, 3 Bunkyo-cho, Matsuyama, Ehime 790-8577, Japan; 2 Department, Molecular Engineering, Graduate School of Engineering, Kyoto University, Katsura, Kyoto 615-8520, Japan; 3 Panasonic Corporation, 1006 Kadoma, Kadoma, Osaka 571-8501, Japan; 4 Department of Chemistry, Graduate School of Science, Kobe University, 1-1 Rokkodai-cho, Nada-ku, Kobe 657-8501, Japan; 5 Elements Strategy Initiative for Catalysts and Batteries (ESICB), Kyoto University, Katsura, Kyoto 615-8520, Japan

**Keywords:** cyclic voltammetry, positive electrode materials, rechargeable battery, redox, tetrathiafulvalene

## Abstract

Derivatives of tris-fused TTF extended with two ethanediylidenes (**5**), tris- and pentakis-fused TTFs extended with two thiophene-2,5-diylidenes (**6**–**9**) were successfully synthesized. Cyclic voltammograms of the tetrakis(*n*-hexylthio) derivative of **5** and **7** (**5d**, **7d**) consisted of two pairs of two-electron redox waves and two pairs of one-electron redox waves. On the other hand, four pairs of two-electron redox waves and two pairs of one-electron redox waves were observed for the tetrakis(*n*-hexylthio) derivative of **9** (**9d**). Coin-type cells using the bis(ethylenedithio) derivatives of **5** (**5b**), **6** (**6b**) and the tetrakis(methylthio) derivatives of **5** (**5c**) and **8** (**8c**) as positive electrode materials showed initial discharge capacities of 157–190 mAh g^−1^ and initial energy densities of 535–680 mAh g^−1^. The discharge capacities after 40 cycles were 64–86% of the initial discharge capacities.

## Introduction

Tetrathiafulvalene (TTF, **1a**) and its analogues have attracted much attention as potential components for organic functional materials as well as multi-electron redox systems [1–5]. Fused TTF oligomers [5] are of considerable interest as multi-electron redox systems, because the TTF units strongly interact with each other. For example, a bis-fused TTF, 2,5-bis(1,3-dithiol-3-ylidene)-1,3,4,6-tetrathiapentalene (BDT-TTP or simply TTP) exhibits four pairs of one-electron redox waves at +0.44, +0.62, +1.05 and +1.13 V (V vs SCE, in benzonitrile) [6]. The *E*_2_–*E*_1_ value is considerably larger than most dimeric TTF derivatives linked by σ-bond (typically 0.05–0.10 V) [7]. Fused TTF donors also play important roles in the development of highly functional materials. For example, TTP and its derivatives have yielded a large number of molecular conductors retaining metallic conductivity down to ≤4.2 K, because they have a tendency to construct two-dimensional molecular arrays through side-by-side sulfur···sulfur interaction [5,8]. On the other hand, a tris-fused TTF, 2,2’-bis[5-(1,3-dithiol-2-ylidene)-1,3,4,6-tetrathiapentanylidene] (TTPY, **2a**) and its derivatives have afforded highly conducting SbF_6_^−^ and iodine salts of σ_rt_ ≈ 10^−1^–10^1^ S cm^−1^ on compressed pellets [9–10].

Recently, we reported that TTP and TTPY can be utilized as positive electrode materials for rechargeable batteries [11]. All organic molecules exhibiting multi-electron redox behaviour seem to be promising as active materials for rechargeable batteries. However, most organic molecules have a crucial disadvantage, that is, they dissolve in organic solvents used for electrolyte solutions. TTF cannot be used as an active electrode material for rechargeable batteries for the above reason, while bis(ethylenedithio)-TTF (BEDT-TTF, **1b**) exhibits relatively good charge–discharge cycle performance because of its lower solubility in organic solvents [12]. However, the substitution of two ethylenedithio groups on TTF results in a significant decrease in the theoretical capacity (about half that of TTF). Utilization of polymerized materials is one of the solutions to decrease solubility. However, insertion of a linkage group, which is usually required to construct polymers, also results in considerable decrease in the theoretical capacity [13]. As for fused TTF oligomers, theoretical capacity rather increases as the number of TTF units increases because two carbons are shared in the two TTF units. TTP and TTPY are actually less soluble in organic solvents than TTF. In particular, TTPY is barely soluble in common solvents even in carbon disulfide. However, the maximum electrons cannot be utilized for TTP and TTPY batteries because TTP and TTPY dissolve in the electrolyte solutions in their maximum oxidation states (tetravalent for TTP and hexavalent for TTPY, respectively) [11].

**Figure 1:**
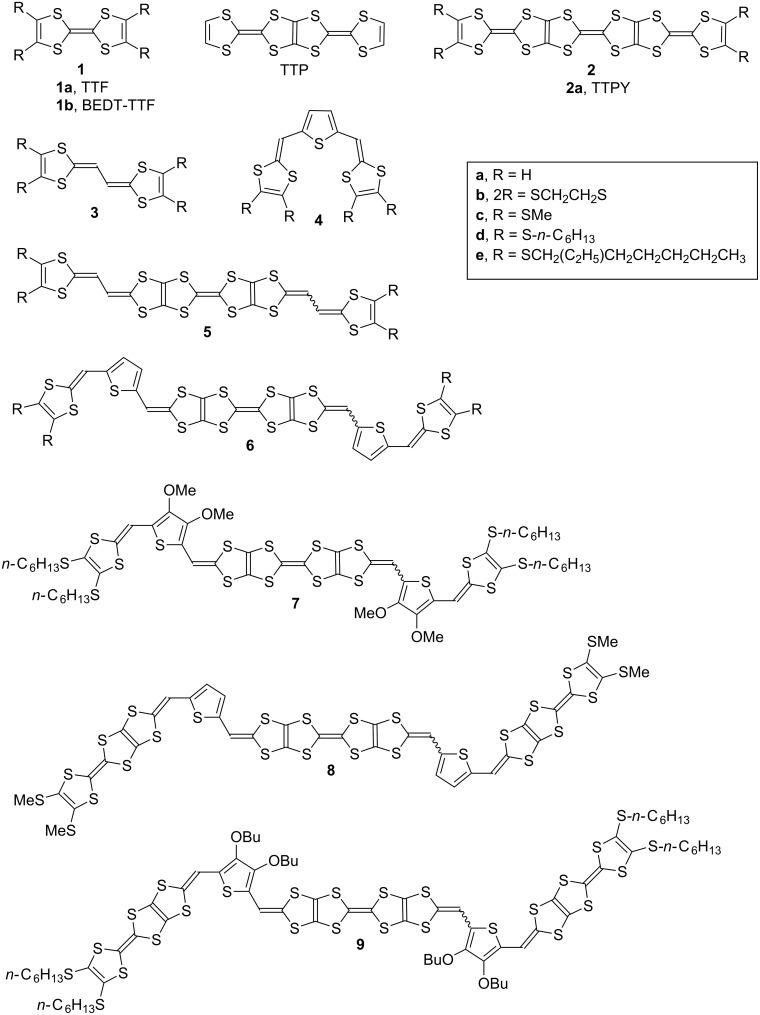
Chemical structures of **1**–**9** and TTP.

Possible molecular modifications for TTPY to reduce solubility in electrolyte solvents are as follows; (i) introduction of rigid substituents such as the ethylenedithio group as mentioned above, (ii) use of a rigid extended-TTF unit, (iii) increase of the number of (extended) TTF units. As for the modification (ii), insertion of a π-spacer is sometimes useful. A vinylogous TTF (**3**, Figure 1) [14–16] shows lower solubility in ordinary organic solvents than TTF, although the thiophene-containing analog (**4**, Figure 1) [17–19] is more soluble than TTF. Increase of the TTF units might be the best way; however, the preparation of tetrakis- and/or pentakis-fused TTFs is not easy because of the low solubility of the precursor molecules. Insertion of thiophene spacers is a possible strategy for synthesizing fused TTF oligomers, because thiophene inserted precursors are more soluble than the TTF-type precursors as mentioned above. We succeeded in the synthesis of fused TTF pentamer and heptamer composed of the unit of **4** [20]. In this paper, we report the synthesis and electrochemical properties of vinyl extended TTPY analogue (**5b**–**d**) and tris- and pentakis-fused TTF analogues extended by the insertion of two thiophene rings (**6b**, **6d**, **7d**, **8c** and **9d**). We also report the charge–discharge properties of rechargeable batteries incorporating the methylthio and ethylenedithio derivatives of **5**, **6** and **8** (**5b**, **5c**, **6b** and **8c**) as a positive electrode material.

## Results and Discussion

### Synthesis

**Scheme 1:**
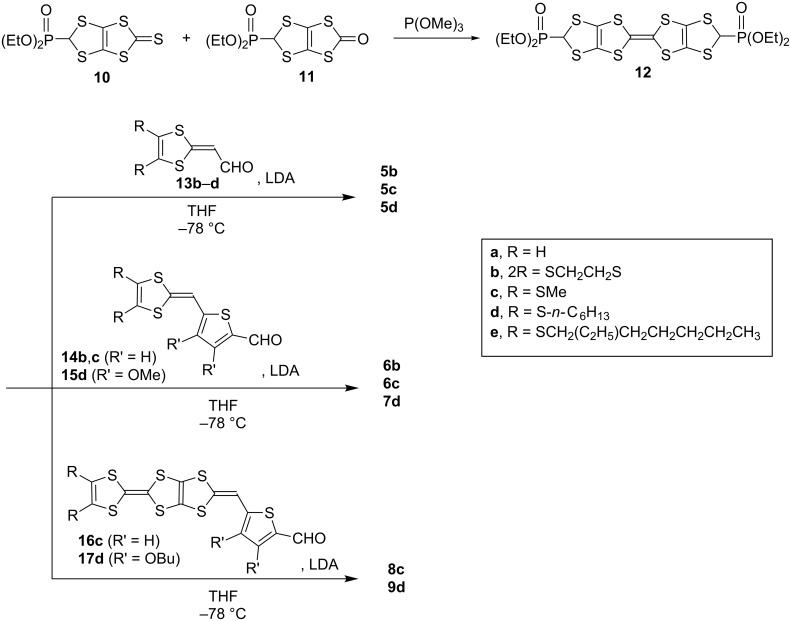
Synthesis of **5**–**9**.

The synthesis of new donors was carried out according to Scheme 1. A trimethylphosphite-mediate cross coupling between a 1,3-dithiole-2-thione (**10**) [21] and 1,3-dithiol-2-one (**11**) [22–24] gave a TTF derivative with two ethoxyphosphoryl groups (**12**) in 63% yield. We adopted the cross-coupling reaction between the 1,3-dithiole-2-thione and the 1,3-dithiol-2-one derivatives for the following reasons. The homo-coupling reaction of **10** afforded **12** in low yields, and purification by column chromatography was difficult because of undesired byproducts. The homo-coupling reaction using **11** might give **12** in a good yield, however, toxic and expensive mercury(II) acetate has to be used for the synthesis of **11**. Thus, the cross-coupling reaction is useful for saving **11**. The Horner–Wadsworth–Emmons reaction of **12** with 2 equiv of aldehydes **13b**–**d** [16,25] in the presence of BuLi in THF at −78 °C gave the desired bis-adducts **5b**–**d** in 54−85% yields. Similarly, **6b**, **6c**, **7d**, **8c** and **9d** were obtained in 62–85% yields by the reaction of **12** with **14b,c**, **15d** [17–19] or **16c**, **17d** [26] in the presence of BuLi in THF. All the new donors were obtained as stable solids.

### Theoretical calculations

**Figure 2:**
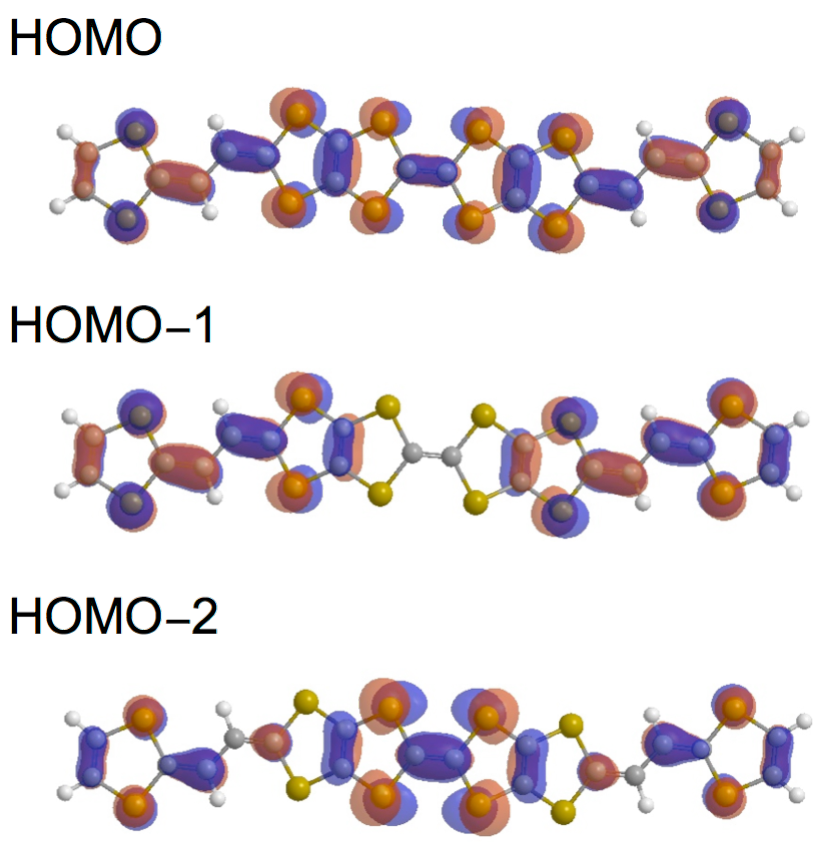
Molecular orbitals of **5a** (*trans* isomer).

**Figure 3:**
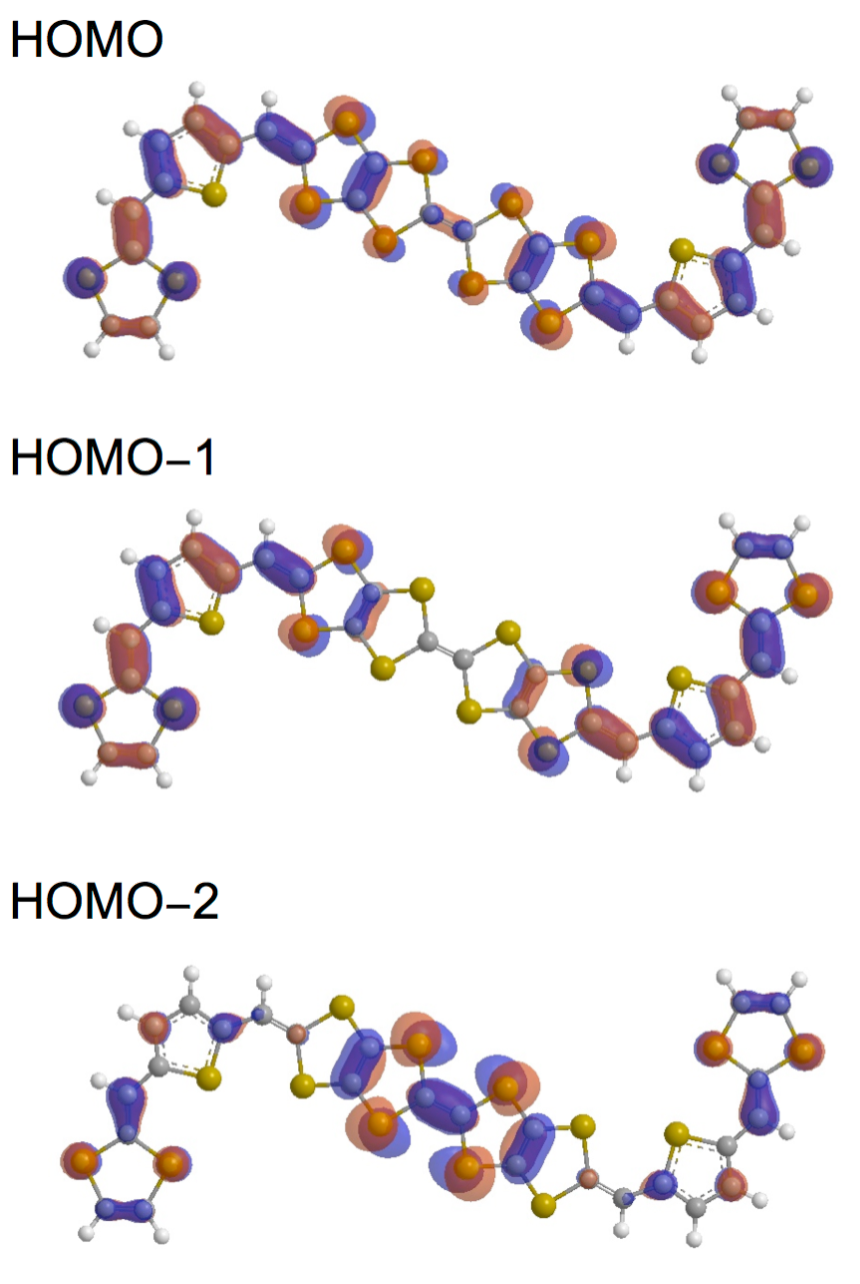
Molecular orbitals of **6a** (*trans* isomer).

**Figure 4:**
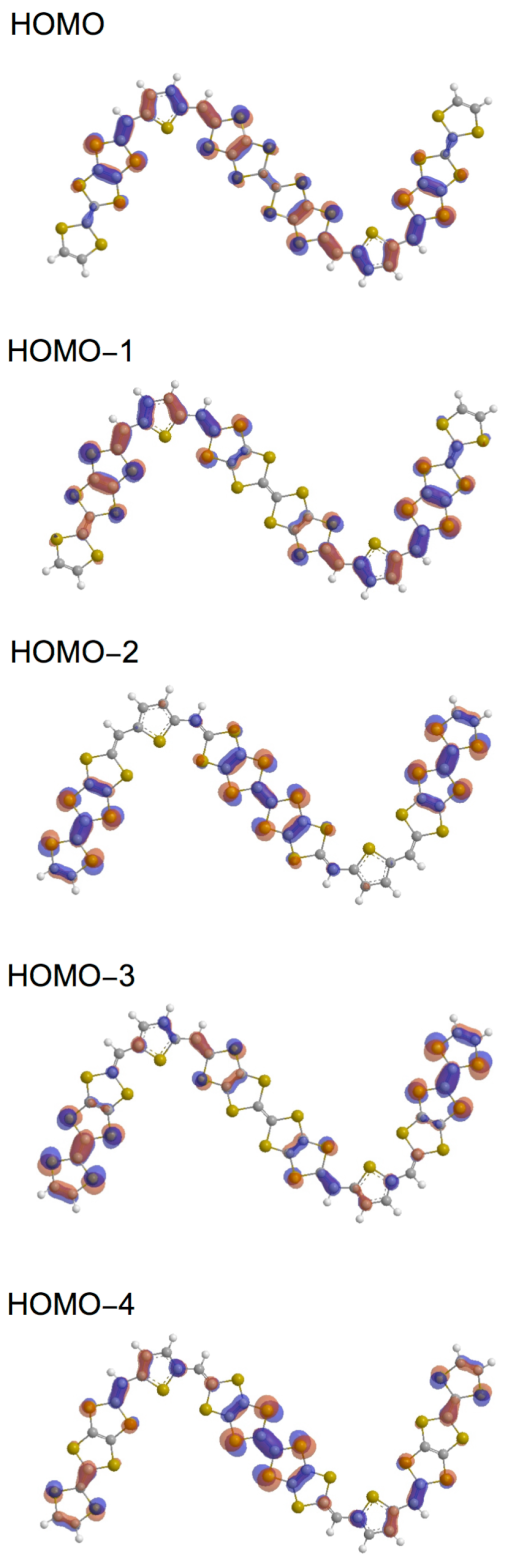
Molecular orbitals of **8a** (*trans* isomer).

We performed theoretical calculations of **5A**, **6Aa** and **7Aa** by using the Gaussian 09 program based on the density functional theory (DFT) at the B3LYP/6-31G(d) level [27]. Their HOMO and HOMO–*n* (*n* = 1–2 and 1–4) of the *trans* isomers of **5a**, **6a** and **8a** are shown in Figures 2–4, respectively. The shapes, energy levels and total energies of the *trans* and *cis* isomers were almost the same as each other. The HOMO of **5a** was distributed over the whole molecules. Molecular orbital coefficients were largely observed in the vinylogous TTF moieties rather than in the central TTF moiety (Figure 2). In the HOMO–1, most molecular orbital coefficients were found on the bilateral vinylogous TTF moieties. The HOMO–2 was mainly located on the central TTF unit. In the bilateral vinylogous TTF moieties, small molecular orbital coefficients were observed. The shapes of the HOMO, HOMO-1 and HOMO-2 of the thiophene extended donor **6a** resembled those of **5a** (Figure 3). The HOMO of **8a** spread mainly over the central TTF and the bilateral extended TTF moieties, and the TTF moieties at the both ends barely contributed to the HOMO (Figure 4). Similarly to **6a**, larger molecular orbital coefficients of the sulfur atoms were found in the extended TTF moieties of **8a** rather than in the central TTF moiety. The HOMO–1 was mainly distributed on the two extended TTF moieties. Small molecular orbital coefficients were observed in the TTF moieties at the both ends. In contrast, the HOMO–2 was hardly distributed on the extended TTF units, but was substantially located on the three TTF units. The TTF moieties at the both ends considerably contributed to the HOMO–3. The HOMO–4 is distributed mainly on the central TTF moiety, although small molecular orbital coefficients were observed in the other donor units.

The orbital energies of the HOMO and HOMO–*n* (*n* = 1–2 and 1–4) for **5a**, **6a** and **8a** are summarized in Table 1. The orbital energies of HOMO of **5a**, **6a** and **8a** (−4.532 to −4.605 eV) are comparable to each other, and are higher by 0.18−0.25 eV than that of TTPY (−4.787 eV). If the oxidation relates to the orbital energy, the first oxidations of **5**–**9** might occur at lower potentials than TTPY. The energy differences between the HOMO and HOMO–1 of all the donors (0.041–0.113 eV) were smaller than that of TTPY (0.186 eV). In particular, the orbital energies of the HOMO and HOMO–1 of **8a** (−4.602 and −4.643 eV, respectively) were close to each other, suggesting that the first four-electron oxidation of **8a** might occur in a narrow potential range. The HOMO–2 of **5a** and **6Aa** (−5.257 and −5.129 eV, respectively) and HOMO–4 of **8a** (−5.328 eV) were slightly higher than the orbital energy of the HOMO–2 of TTPY (−5.439 eV). These results suggest that the electrons at the HOMO–2 of **5a** and **6a** and HOMO–4 of **8a** might be removed more easily than those at the HOMO–2 of TTPY.

**Table 1:** Orbital energies (eV) of **5a**, **6a** and **8a**.

	**5a**	**6a**	**8a**	TTPY

HOMO	−4.605	−4.532	−4.602	−4.787
HOMO–1	−4.718	−4.589	−4.643	−4.973
HOMO–2	−5.257	−5.129	−4.967	−5.439
HOMO–3	–	–	−5.061	–
HOMO–4	–	–	−5.328	–

### Electrochemical properties

**Figure 5:**
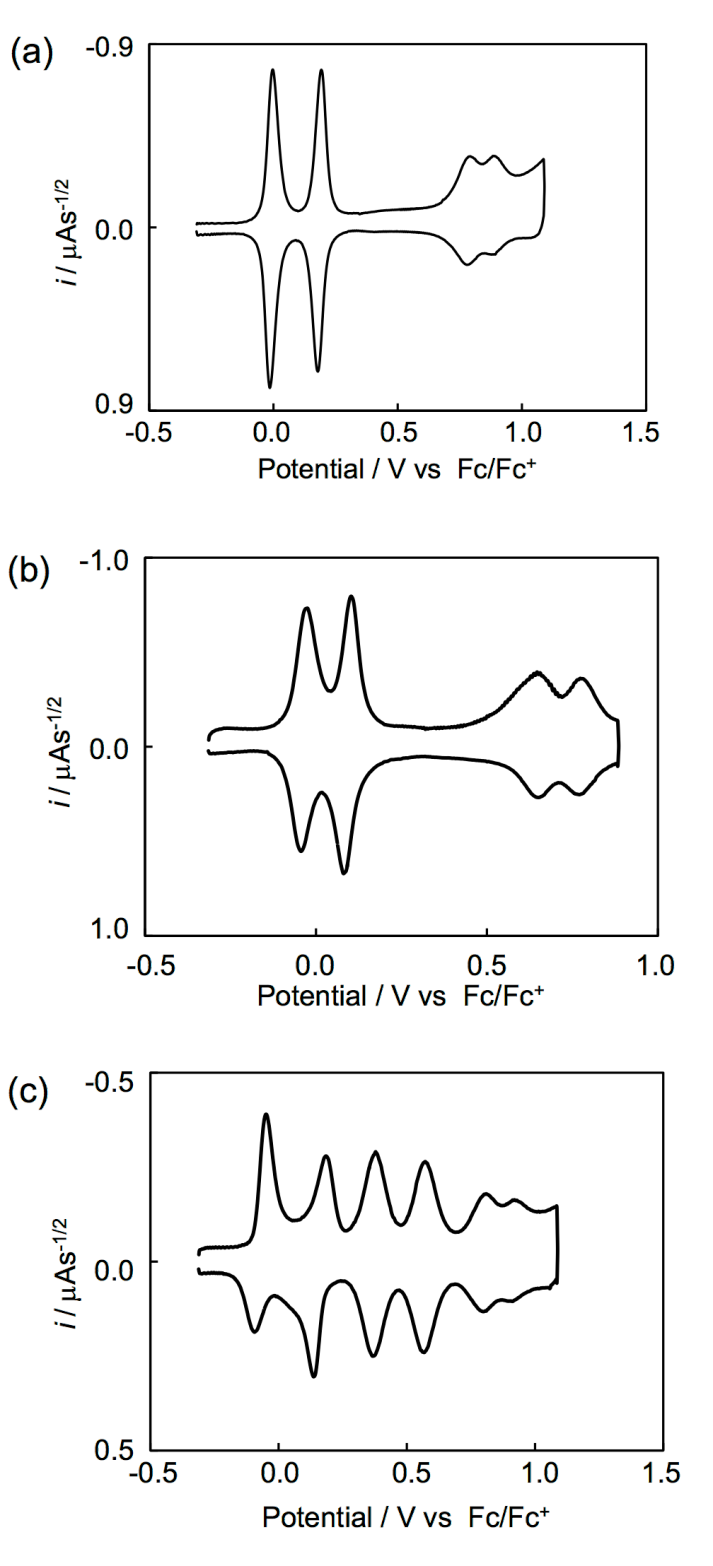
Deconvoluted cyclic voltammograms of (a) **5d**, (b) **7d** and (c) **9d**.

The redox behaviors of **5d**, **7d** and **9d** were investigated by using cyclic voltammetry. Deconvoluted cyclic voltammograms of **5d**, **7d** and **9d** measured in a carbon disulfide/benzonitrile (1:1, v/v) solution are shown in Figure 5. As for the tris-fused donors **5d** and **7d**, four pairs of redox waves were observed. The peak currents of the first and second redox waves were about double those of the others. The maximum number of electrons participating in the redox was six, considering that both donors have six redox-active 1,3-dithiol-2-ylidene (DT) sites. Thus, we think that the first and second redox waves correspond to two-electron redox processes and that the remaining waves correspond to one-electron transfer processes. The pentakis-fused donor **9d** shows six pairs of redox waves. The peak currents of the last two pairs of redox waves were about half as large as those of the others. Considering that **9d** has ten redox-active 1,3-dithiol-2-ylidene sites, it is suggested that the last two pairs of redox waves of **9d** correspond to one-electron transfer process, while the others correspond to two-electron transfer processes.

**Scheme 2:**
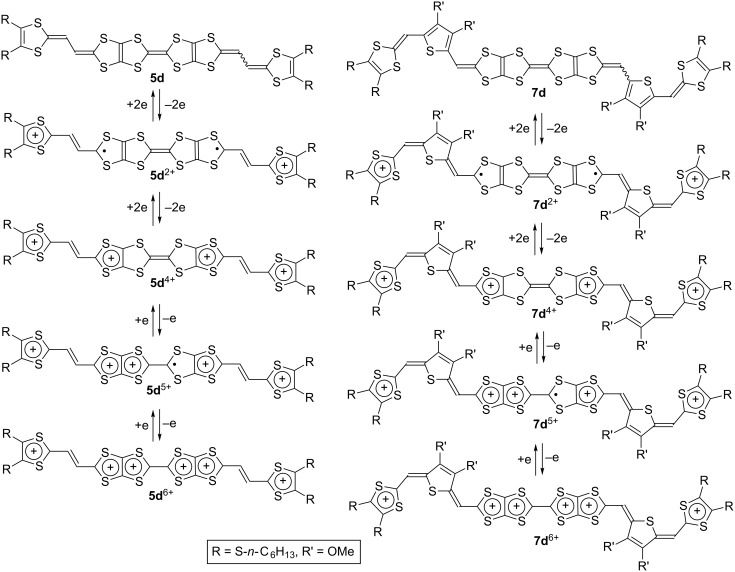
Plausible redox processes of **5d** and **7d**.

The redox potentials of **5d**, **7d** and **9d** are summarized in Table 2 together with their related compounds. The first two-electron redox potentials of **5d** (*E*_m1_ = −0.01 V) and **7d** (*E*_m1_ = −0.04 V) were more negative by 0.13 and 0.16 V than the first redox potential of a TTPY derivative **2e** (*E*_1_ = +0.12 V) measured under the identical conditions. The first redox waves of **5d** and **7d** involved two-electron transfer processes, and that *E*_1_ of the extended donors **3c** and **18** (−0.06 V) was lower than that of the TTF derivative **1c** (+0.03 V). These results suggest that two positive charges formed by the first two-electron oxidation process of **5d** and **7d** are presumably distributed mainly on each of the two extended TTF moieties so as to reduce on-site Coulomb repulsion (Scheme 2). Similarly, the two positive charges in **5d**^4+^ and **7d**^4+^ might be located mainly on each of the two extended TTF donors. Observation of two sequent one-electron redox waves in the higher potential region (+0.6 to +0.9 V) indicates that the central TTF moiety contributes to the remaining redox processes. The significant positive shifts of the *E*_5_ and *E*_6_ of **5d** and **7d** by 0.62–0.75 V and 0.41–0.63 V, respectively, compared to the *E*_1_ and *E*_2_ of **3c** is probably due to the strong electron-withdrawing effect by two dicationic extended TTF units in the tetracationic states. In other words, the presence of five and six positive charges in the molecules induces significantly large on-site coulomb repulsion. The *E*_5_ and *E*_6_ of **7d** are lower by 0.12–0.14 V than those of **5d**, suggesting that **7d**^6+^ is more stabilized than **5d**^6+^. The thiophene spacers inserted between two cationic 1,3-dithiole rings might reduce the intramolecular coulomb repulsion in **7d**^6+^.

**Table 2:** Redox potentials of **5d**, **7d**, **9d** and their related compounds (V vs Fc/Fc^+^, in benzonitrile/carbon disulfide 1:1, v/v).

Donor	*E*_1_	*E*_2_	*E*_3_	*E_4_*	*E*_5_	*E*_6_	*E*_7_	*E*_8_	*E*_9_	*E*_10_
	*E*_m1_^a^		*E*_m2_^a^		*E*_m3_^a^		*E*_m4_^a^			

**5d**	−0.01		+0.19		+0.79	+0.89				
**7d**	−0.04		+0.09		+0.65	+0.77				
**9d**	−0.07		+0.16		+0.38		+0.57		+0.81	+0.92
**2e**	+0.12	+0.19	+0.39		+0.87					
**1c**	+0.03	+0.36								
**3c**	−0.06	+0.05								
**18**	−0.06	+0.02								
**19**	+0.03		+0.36	+0.56						

^a^* E*_m1_ = (*E*_1_+*E*_2_)/2. *E*_m2_ = (*E*_3_+*E*_4_)/2. *E*_m3_ = (*E*_5_+*E*_6_)/2. *E*_m4_ = (*E*_7_+*E*_8_)/2.
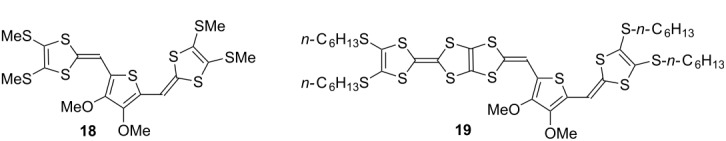

**Scheme 3:**
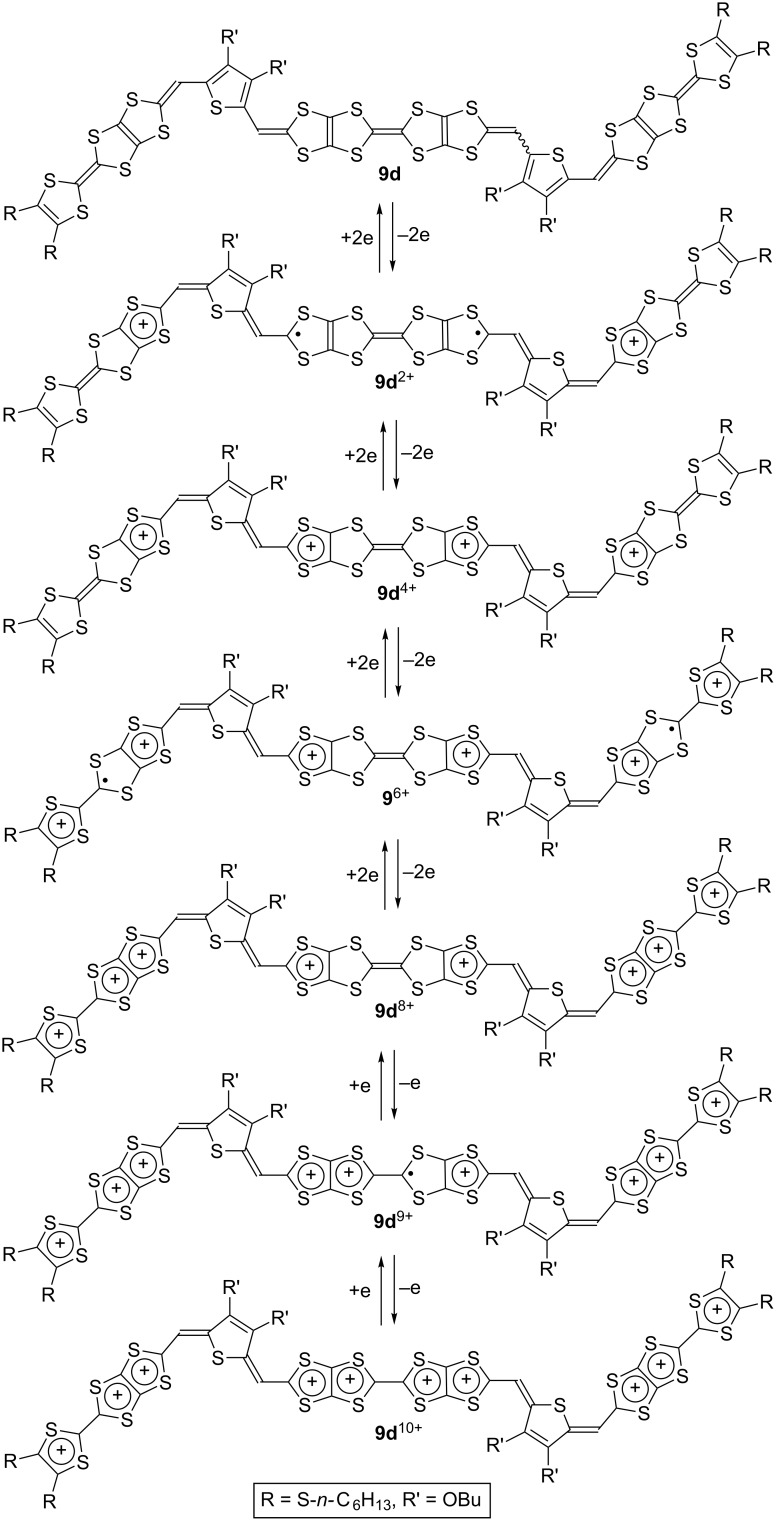
Plausible redox process of **9d**.

As for **9d**, two positive charges in **9d**^2+^ are probably distributed mainly on each of the two thiophene-inserted TTF moieties, since the first redox wave of **9d** corresponds to a simultaneous two-electron transfer process, and the *E*_m1_ (−0.07 V) is comparable to the *E*_1_ of **18** (−0.06 V) as shown in Scheme 3. The *E*_m2_ of **9d** (+0.16 V) is lower by 0.20 V than the *E*_3_ of **19** (+0.36 V) [28], suggesting that the second redox process is contributed by two extended TTF moieties similarly to the first redox process. On the other hand, both the third and fourth redox waves involve two-electron transfer, and their potentials (*E*_m3_ = +0.38 V, *E*_m4_ = +0.57 V, respectively) are comparable to the *E*_3_ and *E*_4_ of **19** (*E*_3_ = +0.36 V, *E*_4_ = +0.56 V, respectively). Therefore, it is indicated that positive charges formed by the third and fourth redox processes are distributed over two TTF moieties at the both ends. The central TTF moiety contributes to the remaining two stages of the one-electron redox processes at +0.81 and +0.92 V, similarly to **5d** and **7d**.

### Charge and discharge properties of rechargeable batteries

In order to examine the cell performance, IEC R2016 coin-type cells were fabricated using a positive electrode incorporating **5**, **6** and **8** (**5b, 5c, 6b** and **8c**) as positive electrode materials. The applied current densities were 40 mA g^−1^ (0.2 C rate) and 100 mA g^−1^ (0.5 C rate), respectively. The nominal charge capacity of a battery or an electrode is expressed as a C-rate. A 0.2 C rate means that the full discharge capacity reached in 5 h. Cyclic voltammetry in the solid state was carried out prior to the charge–discharge test so as to determine the turning back voltage (Supporting Information File 1, Figure S4). The electrodes incorporating **5b** and **5c** exhibited three indistinct oxidation peaks at 3.4, 3.6 V and 4.1 V. Multi-scan cycle voltammetry revealed a significant decay of redox waves at 4.1 V presumably due to dissolution of the oxidative species of **5b** and **5c** in the electrolyte solution. In contrast, there was no distinct decay of the redox waves at 3.6 V, suggesting that the oxidative species of **5b** and **5c** formed at 3.6 V barely dissolved in the electrolyte solution. In contrast, no distinct dissolution was observed for the **6b** and **8c** cells even at 4.2 V. Thus, we determined the turn-back voltages as 3.8 V for the **5b** and **5c** cells, and 4.2 V for the **6b** and **8c** cell.

**Figure 6:**
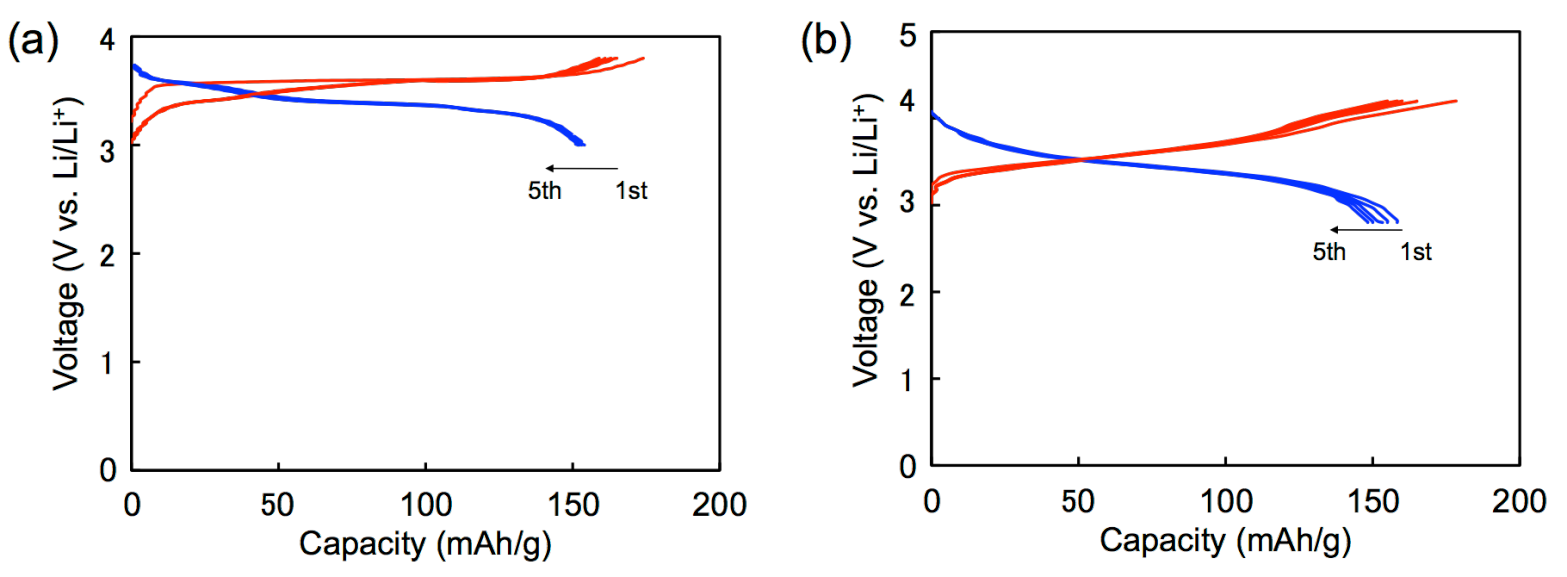
(a) Galvanostatic charge-discharge curves of (a) **5c**/Li and (b) **6b**/Li cells.

**Figure 7:**
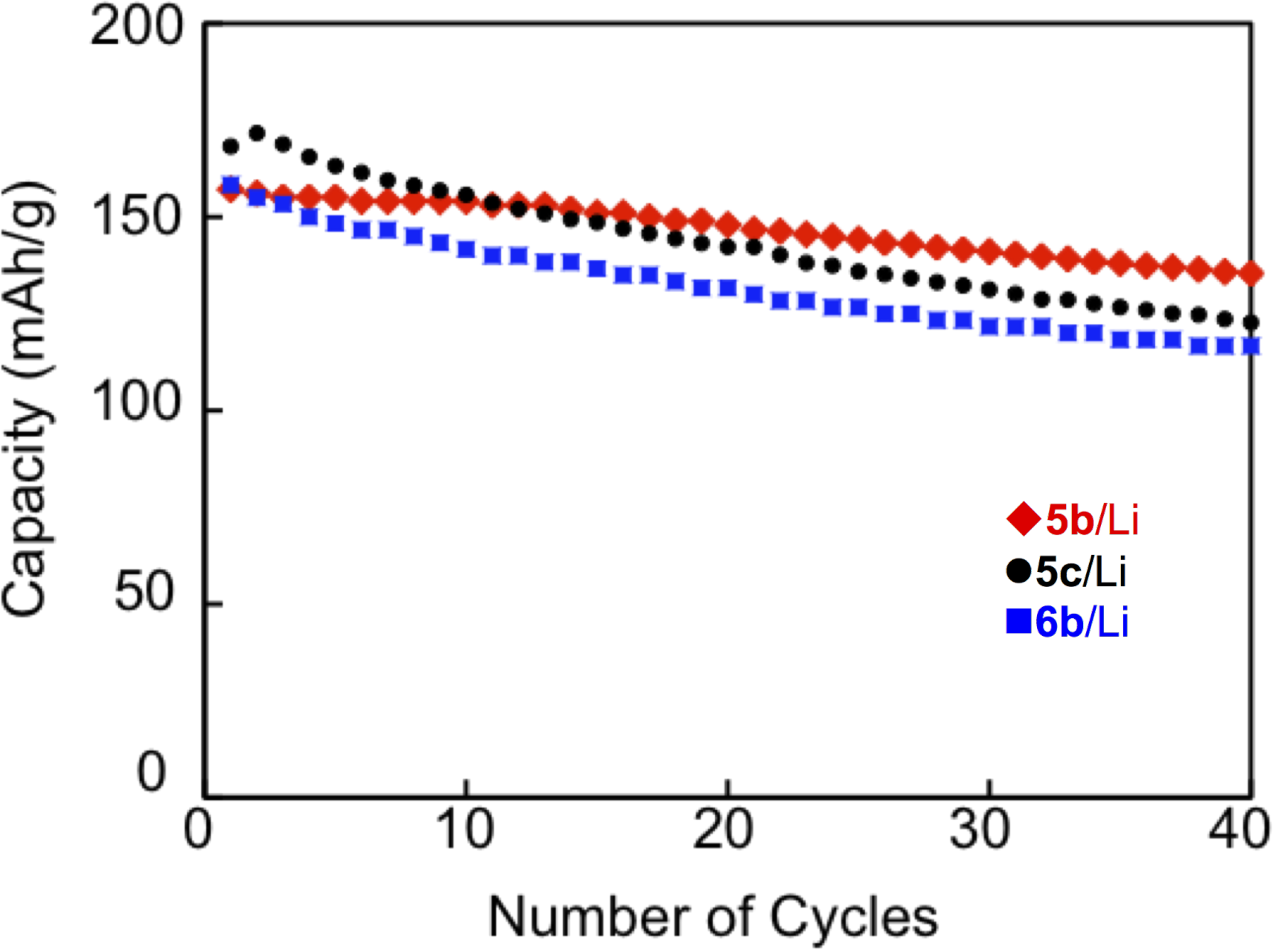
Cycle-life performances for **5b**/Li, **5c**/Li and **6b**/Li cells.

The results are summarized in Table 3, and the first five charge–discharge curves of **5b**/Li and **6b**/Li cells cycled at room temperature are shown in Figure 6. A **5c** cell also exhibited charge–discharge curves similar to **5b**/Li and **6b**/Li cells. No distinct plateau was observed in both the charge and discharge processes for all the cells in spite of observation of well-separated redox waves in a solution. This is possibly due to the apparent overlap of the redox processes in the solid state (see also Figure S4 in Supporting Information File 1) [11]. The initial discharge capacities of **5b**/Li, **5c**/Li and **6b**/Li cells were 157, 168 and 158 mAh g^−1^, respectively. They correspond to 93 and 99% of the theoretical capacity for the five-electron redox of **5b** and **5c**, and 94% of the theoretical capacity for the six-electron redox of **6b**, respectively. The first discharge capacities observed are comparable to those of the positive active materials for commercially available lithium ion batteries on the market (150–170 mAh g^−1^). Cycle-life performances for **5b**/Li, **5c**/Li and **6b**/Li cells are shown in Figure 7. In all cases, the discharge capacities decreased gradually as the number of cycles increased. The discharge capacities after 40 cycles were 86, 73 and 74% of the first discharge capacities for **5b**/Li, **5c**/Li and **6b**/Li cells, respectively. The decrease in capacities for the cells using organic electrode materials might be attributed to elution of the positive electrode materials into the electrolyte solution. The result that the **5b** cell shows higher cycle performance than the other cells is consistent with the lower solubility of **5b** with rigid ethylenedithio substituents than **5c** with flexible methylthio substituents.

**Table 3:** Charge–discharge parameters for the rechargeable batteries using **5**, **6** and **8**.

	**5b**/Li	**5c**/Li	**6b**/Li	**8c**/Li

Theoretical capacities for maximum electrons utilization indicated in parentheses (mAh g^−1^).	203(6)	203(6)	169(6)	205(10)
1^st^ Discharge capacity (mAh g^−1^)	157	168	158	190
Number of electron per molecule participating discharge	5	5	6	10
Average voltage for 1^st^ discharge (V)	3.41	3.40	3.44	3.58
1^st^ Energy density (mWh g^-1^)	535	571	544	680
40^th^ Discharge capacity/1^st^ discharge capacity (%)	86	73	74	64

**Figure 8:**
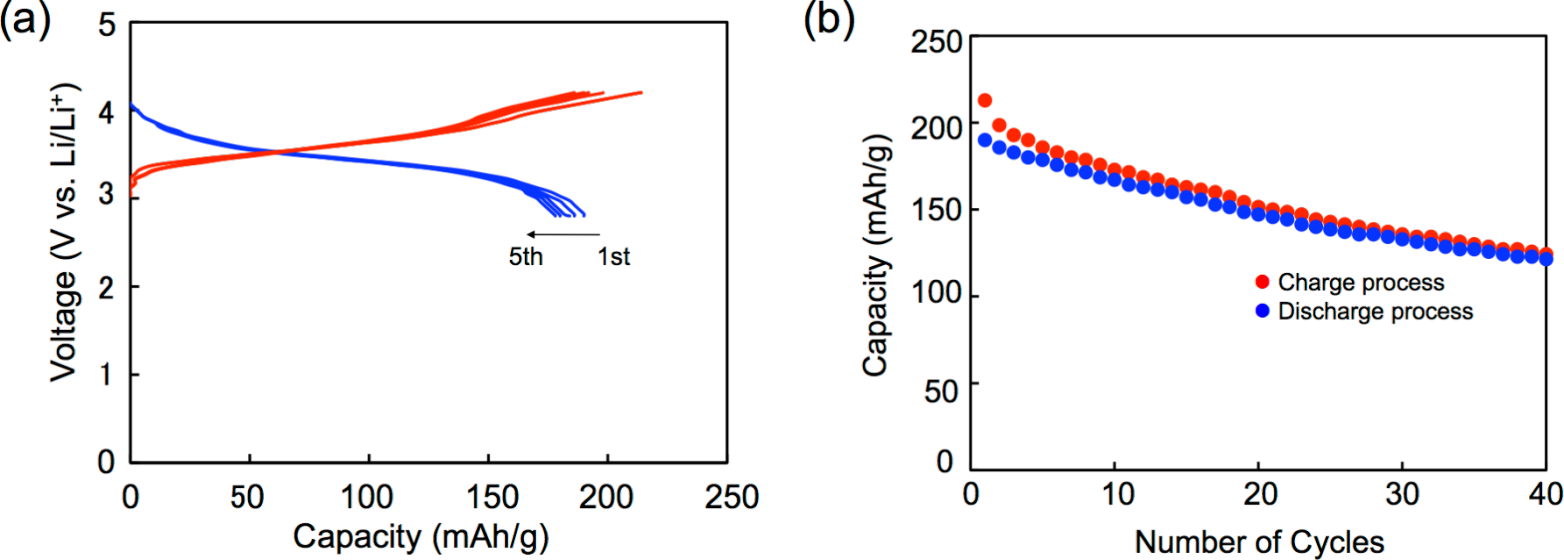
(a) Galvanostatic charge–discharge curves, and (b) cycle-life performances for a **8c**/Li cell.

Figure 8 shows the first five charge–discharge curves of a **8c**/Li cell cycled at room temperature and their cycle performances up to 40 cycles. The initial charge and discharge capacities were 202 and 190 mAh g^−1^, respectively. They correspond to 99% and 93% of the theoretical capacity for ten-electron utilization (205 mAh g^−1^). This result strongly indicates that ten-electron redox per molecule participates in the charge and discharge processes. Similarly to the **5b**, **5c** and **6b** cells, the **8c**/Li cell shows no distinct plateau in both the charge and discharge processes in spite of observation of six pairs of redox waves in a solution. The initial discharge capacity of the **8c**/Li cell (190 mAh g^−1^) is higher than those of the cathode active materials for commercially available lithium ion batteries. The discharge capacity after 40 cycles (121 mAh g^−1^) was 64% of the first discharge capacity. The high cycle-life performance in spite of utilization of the highest oxidation state of +10 might be because of strong van der Waals force between the large π-electron frameworks of the thiophene-inserted pentakis-fused TTF.

**Figure 9:**
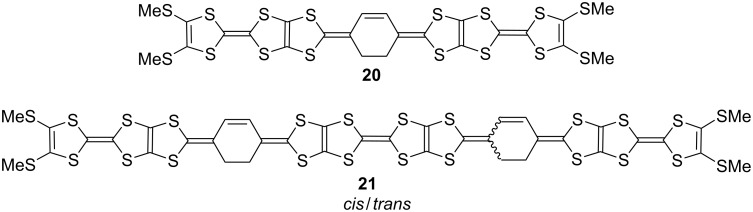
Molecular structures of **20** and **21**.

The energy densities for the first discharge process (1^st^ energy densities) calculated by multiples of the initial discharge capacity and the average voltage are also summarized in Table 3. The first energy densities of **5b**, **5c** and **6b** cells were 535–571 mWh g^−1^, which are comparable to that of TTPY (543 mWh g^−1^ for four-electron utilization). On the other hand, the first energy density of **8c** cell (680 mWh g^−1^) is larger which is larger by 110–150 mWh g^−1^ than those of the others. This value is also superior to the energy densities of most inorganic cathode materials for LIBs [29–30], **20**/Li (605 mWh g^−1^) [31], and is slightly smaller than that of **21**/Li (700 mWh g^−1^) [32] (Figure 9).

## Conclusion

A TTF derivative with two phosphonate groups (**12**) is a useful building block for the synthesis of odd-numbered fused TTF donors containing extended TTF units. We have demonstrated that some derivatives of **5**, **6** and **8** can be utilized as positive electrode materials for rechargeable batteries. The **5b**/Li cell showed considerably higher cycle performance when the number of electrons is suppressed to five per molecule (5/6 of the maximum electrons). It is noted that the **6b** and **8c** cells showed good cycle performance in spite of utilizing the maximum amounts of electrons (six and ten electrons, respectively). The **8c** cell exhibited significantly high energy density (680 mWh g^−1^) at the first discharge thanks to ten electrons utilization. The information obtained from the present work could be helpful in the molecular design and synthesis of new positive electrode materials for rechargeable batteries. We are engaged in the synthesis of the other derivatives of **5**, **6** and **8** containing unsubstituted derivatives and higher homologues of **5**, which are expected to exhibit higher charge–discharge performance than the materials described in this paper.

## Supporting Information

File 1 Experimental details and spectroscopic data, optimized structures of **5a**, **6a** and **8a** (*trans* isomers) and cycle-life performances for rechargeable batteries using **5c**, **5b** and **6b**.
