# Supplementary material for: New tris- and pentakis-fused donors containing extended tetrathiafulvalenes: New positive electrode materials for rechargeable batteries
Source: Beilstein J Org Chem. 2015 Jul 8;11:1136–47. doi: 10.3762/bjoc.11.128 (PMC4505304; doi:10.3762/bjoc.11.128)
Supplement: File 1 — Experimental details and spectroscopic data, optimized structures of 5a, 6a and 8a (trans isomers) and cycle-life performances for rechargeable batteries using 5c, 5b and 6b. [file Beilstein_J_Org_Chem-11-1136-s001.pdf]

Supporting Information
for

New tris- and pentakis-fused donors containing extended tetrathiafulvalenes: New positive electrode materials for rechargeable batteries

Shintaro Iwamoto[1,2], Yuu Inatomi[1,3], Daisuke Ogi[1], Satoshi Shibayama[1], Yukiko Murakami[1], Minami Kato[1], Kazuyuki Takahashi[4], Kazuyoshi Tanaka[2], Nobuhiko Hojo[3] and Yohji Misaki*[1,5]

Address: [1]Department of Applied Chemistry, Graduate School of Science and Engineering, Ehime University, 3 Bunkyo-cho, Matsuyama, Ehime 790-8577, Japan, [2]Department, Molecular Engineering, Graduate School of Engineering, Kyoto University, Katsura, Kyoto 615-8520, Japan, [3]Panasonic Corporation, 1006 Kadoma, Kadoma, Osaka 571-8501, Japan, [4]Department of Chemistry, Graduate School of Science, Kobe University, 1-1 Rokkodai-cho, Nada-ku, Kobe 657-8501, Japan and [5]Elements Strategy Initiative for Catalysts and Batteries (ESICB), Kyoto University, Katsura, Kyoto 615-8520, Japan

Email: Yohji Misaki - misaki.yohji.mx@ehime-u.ac.jp
*Corresponding author

Experimental details and spectroscopic data, optimized structures of 5a, 6a and 8a (*trans* isomers) and cycle-life performances for rechargeable batteries using 5c, 5b and 6b.

Contents

1. Experimental details s2
2. Theoretical calculations for **5a**, **6a** and **8a** (Figures S1–S3). s6
3. Cyclic voltammograms of positive electrodes (Figure S4) s8
4. Cycle-life performances for rechargeable batteries using **5b**, **5c** and **6b** (Figure S5) s9

1. Experimental details

General

^{1}H NMR spectra were recorded on JEOL NM-SCM270 or JEOL NM-EX400 instrument. Spectra are reported (in δ) with referenced to Me_4Si. $CDCl_3$ was used as solvent. MS spectra were determined on an Applied Biosystem MALDI TOFMS Voyager-DE™ PRO. Only the more intense or structurally diagnostic mass spectral fragment ion peaks are reported. Melting points were determined with a Yanaco MP-J3. Cyclic voltammetries (CV) were recorded on an ALS/chi 617B Electrochemical analyzer. The CV cell consisted of the Pt working electrode, Pt wire counter electrode, and an $Ag/AgNO_3$ reference electrode. The measurements were carried out in carbon disulfide/benzonitrile (2:1, v/v) solution of samples with a concentration of 0.1 M n-$Bu_4N{\bullet}PF_6$ as a supporting electrolyte. All redox potentials were measured against Ag/Ag^+ and converted to vs. Fc/Fc^+. The cyclic performance of the fabricated cell was examined by repeated charging–discharging galvanostatic cycles at different current densities using NAGANO-TS2005W.

Preparation of electrodes and battery cells

In a similar manner as described in [11], the positive electrodes were prepared by mixing of the active material (**5b,c**, **6b**, **8c**, 20% weight), acetylene black (Denki Kagaku Kogyo) as the conductive additive (65%), and poly(tetrafluoroethylene) as the binder (15%). A mixed solution of ethylene carbonate and diethyl carbonate (1:5, v/v) containing 1.0 M $LiBF_4$ was used as an electrolyte solution. A coin cell was fabricated by sandwiching the electrolyte layer of electrolyte solution with the cathode and the Li anode, using the separator film under dried air conditions.

Electrochemical analyses of the positive electrodes and cells

In a similar manner as described in [11], electrochemical analyses were carried out in a conventional cell under dried air. The auxiliary electrode and reference electrode was a Li sheet. A Solartron 1287 Electrochemical analyzer was employed to obtain the voltammograms and charge-discharge cycle test. The cyclic performance of the fabricated cell was examined by repeated charging–discharging galvanostatic cycles at different current densities. The charging-discharging experiments were typically performed at a current density of 40 mA g^{-1} (0.2 C) for charge and 100 mA g^{-1} (0.5 C) for discharge processes.

Synthesis of 12

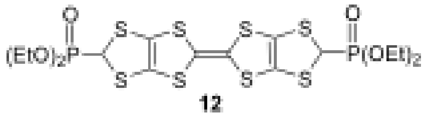

A mixture of **10** (3.1 g, 9.1 mmol) [21] and **11** (3.0 g, 9.1 mmol) [22-24] in trimetylphosphite (35 ml) and toluene (50 ml) was stirred at 110 °C for 2 h under argon atmosphere. After cooling to room temperature, hexane was added. The resulting precipitate was collected, washed with hexane, and then subjected to column chromatography on silica gel with dichloromethane/ethyl acetate (1:1, v/v). The crude product was reprecipitated from dichloromethane/hexane to give **12** (3.6 g, 5.7 mmol) as orange powder in 63% yield. m.p. 155.0-156.9 ℃; ^{1}H NMR (270 MHz, C_6D_6, 25 °C, TMS): δ 5.17 (d, *J*=9.2 Hz, 1H), 5.06 (d, *J*=9.5 Hz, 1H), 4.00–4.11 (m, 8H), 1.3 (t, *J*=7.0 Hz, 12H); IR (KBr): 2979, 2898, 1252, 1050, 1024 cm^{-1}; HR-MS (LDI-TOF): *m/z* found 627.8674 (M$^+$), calcd for $C_{16}H_{22}O_6P_2S_8$ 627.8657.

Synthesis of 5c

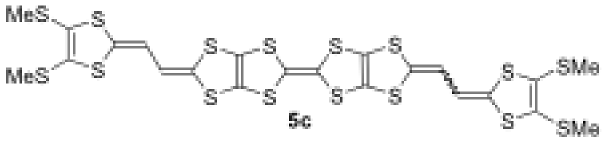

In a similar manner as described in [32], to a solution of **12** (150 mg, 0.24 mmol) and **13c** (125 mg, 0.53 mmol) [25] in THF (20 ml) was added a 0.5 M THF solution of LDA (1.16 ml, 0.58 mmol) at –78 °C. After the reaction mixture was stirred for 1 h, the reaction mixture was warmed up to –50 °C. After methanol (60 ml) was added to the reaction mixture, the resultant precipitation was filtered, and washed with methanol. The obtained solid was dried over *in vacuo* to afford **5c** (142 mg, 0.18 mmol) as in ocher powder 75% yield. ocher powder; m.p. 220–221 ℃; IR (KBr): 2988, 2913, 1532, 1510, 1490, 1426, 1268 cm^{-1}; HR-MS (LDI-TOF): *m/z* found 791.6829 (M$^+$), Calcd for $C_{22}H_{16}S_{16}$ 791.6783.

Synthesis of 5b

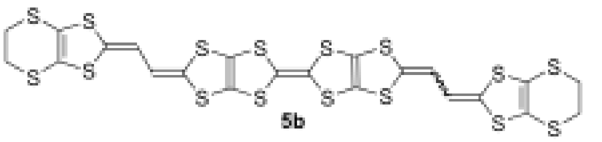

5b

Compound **5b** was obtained from **12** (150 mg, 0.24 mmol) and **13b** (124 mg, 0.53 mmol) in 85% yield (160 mg, 0.20 mmol) in a similar manner as described for **5c**. **5b**: dark brown powder; m.p. 253–254 °C; IR: 2911, 2915, 1676, 1500, 1405 cm^{-1}; HR-MS (LDI-TOF): *m/z* found 787.6489 (M^+), Calcd for $C_{22}H_{12}S_{16}$ 787.6470.

Synthesis of 5d

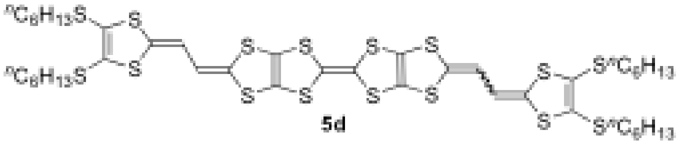

5d

Compound **5d** was obtained from **12** (150 mg, 0.24 mmol) and **13d** (191 mg, 0.53 mmol) in 54% yield (142 mg, 0.13 mmol) in a similar manner as described for **5c**. **5d**: ocher powder; ocher powder; m.p.162.2–164.0°C; ^{1}H NMR (400 MHz, C_6D_6, 25 °C, TMS): δ 5.79 (d, *J* = 10.4 Hz, 2H), 5.62 (d, *J* = 10.4 Hz, 2H), 2.65–2.69 (m, 8H), 1.51–1.57 (m, 8H), 1.26–1.34 (m, 24H), 0.88 (t, *J* = 6.80 Hz, 12H); IR (KBr): 2954, 2924, 2853, 1533, 1510, 1490, 1459 cm^{-1}; HR-MS (LDI-TOF): *m/z* found 1071.9952 (M^+), calcd for $C_{42}H_{56}S_{16}$ 1071.9913.

Synthesis of 6b

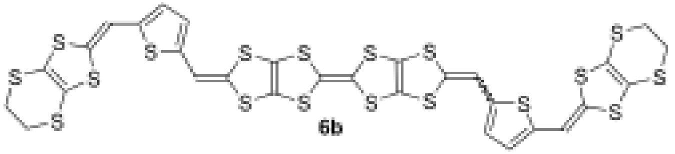

6b

Compound **6b** was obtained from **12** (102 mg, 0.16 mmol) and **14b** (151 mg, 0.48 mmol) in 63% yield (96 mg, 0.10 mmol) in a similar manner as described for **5c**. **6b**: red brown powder; m.p. >300°C; IR (KBr): 3063, 2989, 2916, 1653, 1560, 1518, 1283, 1226, 1049 cm^{-1}; HR-MS (LDI-TOF): *m/z* found 951.6175(M^+), calcd for $C_{30}H_{16}S_{18}$ 951.6225.

Synthesis of 6c

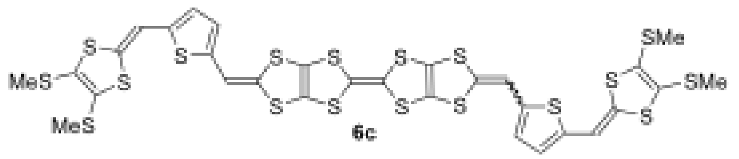

Compound **6c** was obtained from **12** (150 mg, 0.24 mmol) and **14c** (168 mg, 0.35 mmol) in 75% yield (172 mg, 0.18 mmol) in a similar manner as described for **5c**. **6c**: red brown powder; m.p.161–163 °C; ^{1}H NMR (270 MHz, C_6D_6, 25 °C, TMS): δ 6.64 (s, 2H), 6.53 (s, 2H), 6.43 (s, 2H), 2.31 (s, 12H); IR (KBr): 3060, 2989, 2952, 2915, 2850, 1567, 1428 cm^{-1}; HR-MS (LDI-TOF): *m/z* found 955.6440 (M^+), calcd for $C_{30}H_{20}S_{18}$ 955.6538.

Synthesis of 7d

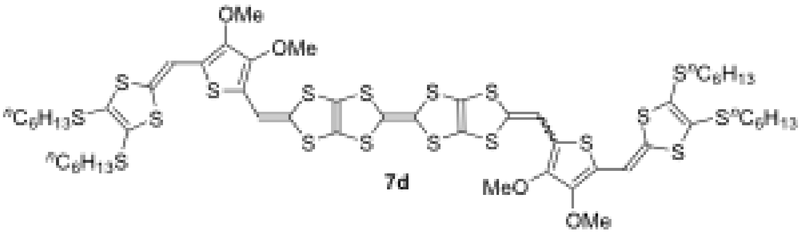

Compound **7d** was obtained from **12** (30 mg, 0.048 mmol) and **15d** (50 mg, 0.097 mmol) in 85% yield (55 mg, 0.041 mmol) in a similar manner as described for **5c**. **7d**: ocher powder; m.p. 65.5–70.0 °C; ^{1}H NMR (270 MHz, $CDCl_3$, 25 °C, TMS): δ 6.79 (s, 2H), 6.69 (s, 2H), 3.85 (s, 6H), 3.83 (s, 6H), 2.80-2.90 (m, 8H), 1.63-1.71 (m, 8H), 0.88-1.45 (m, 24H), 0.90 (t, *J* = 6.48 Hz, 12H); IR (KBr): 2953, 2925, 2854, 1575, 1466, 1399, 1294, 1054 cm^{-1}; HR-MS (LDI-TOF): *m/z* found 1356.1409 (M^+), calcd for $C_{54}H_{68}O_4S_{18}$ 1356.0090.

Synthesis of 8c

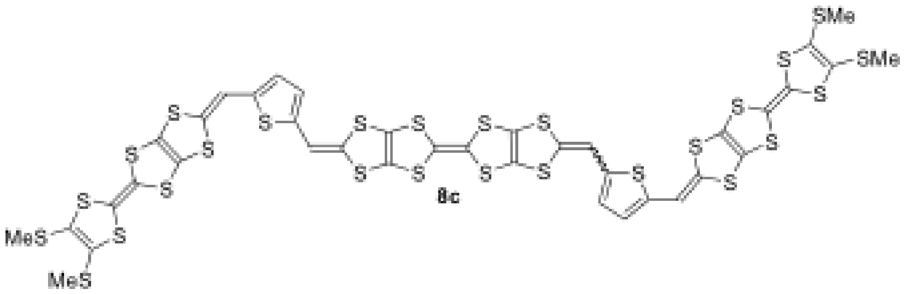

Compound **8c** was obtained from **12** (60 mg, 0.095 mmol) and **16c** (104 mg, 0.21 mmol) in 62% yield (77 mg, 0.059 mmol) in a similar manner as described for **5c**. **8c**: red brown; m.p. >300℃; IR (KBr): 3062, 2990, 2915, 1568, 1509, 1426, 1043 cm^{-1}; HR-MS (LDI-TOF): *m/z* found 1307.4324 (M^+), calcd for $C_{38}H_{20}S_{26}$ 1307.4303.

Synthesis of 9d

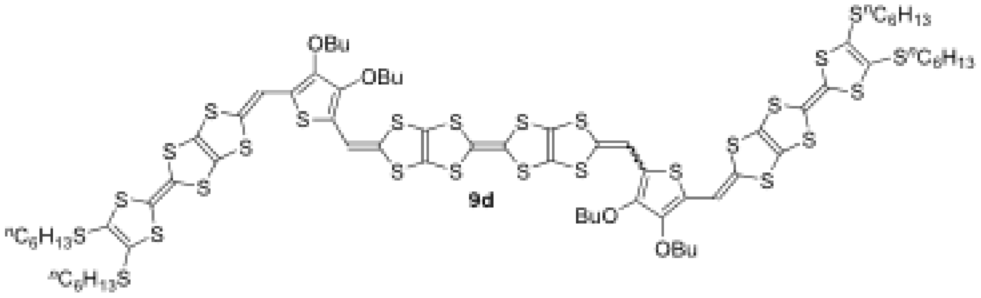

Compound **9d** was obtained from **12** (74 mg, 0.16 mmol) and **17d** (183 mg, 0.24 mmol) in 28% yield (63 mg, 0.034 mmol) in a similar manner as described for **5c**. **9d**: red brown; m.p. 98–101 °C; ^{1}H NMR (400 MHz, $CDCl_3$, 25 °C, TMS): δ 6.693 (s, 1H), 6.689 (s, 1H), 6.68 (s, 2H), 3.880 (t, J = 6.6 Hz, 4H), 3.876 (t, J = 6.6 Hz, 4H), 2.72 (t, J = 6.80 Hz, 4H), 2.71 (t, J = 6.80 Hz, 4H), 1.54–1.64 (m, 16H), 1.27–1.44 (m, 32H), 0.94 (t, J = 7.4 Hz, 12H), 0.89 (t, J = 7.0 Hz, 12H); IR (KBr): 2954, 2925,2868, 1445, 1374, 1291, 1065 cm^{-1}; HR-MS (LDI-TOF): *m/z* found 1875.9711(M^+), calcd for $C_{74}H_{92}O_4S_{26}$ 1875.9734.

2. Theoretical calculations for 5a, **6a** and **8a.**

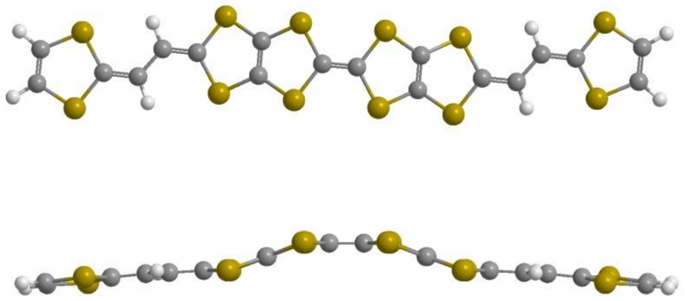

Figure S1. Optimized structures of **5a** (trans isomer).

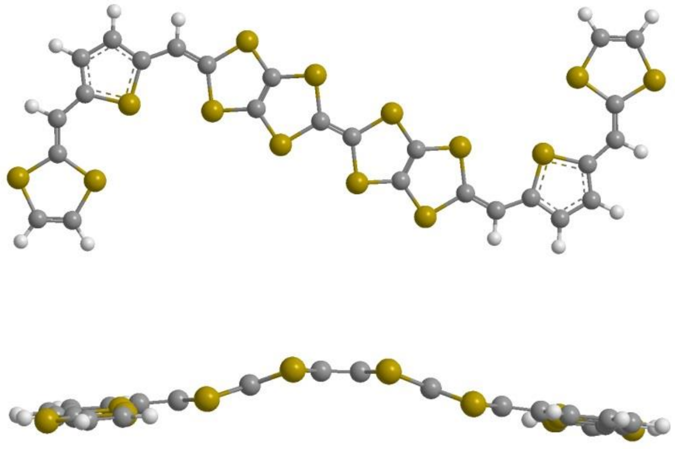

Figure S2. Optimized structures of **6a** (trans isomer).

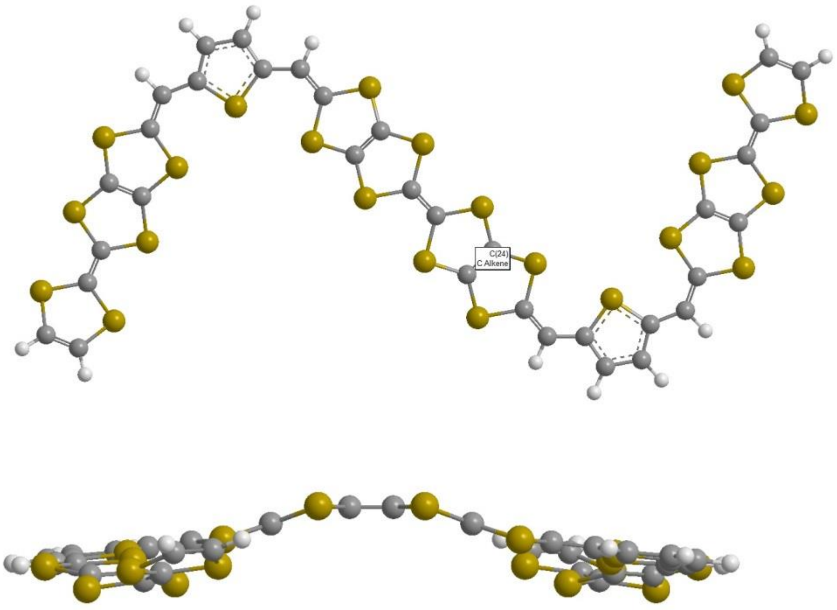

Figure S3. Optimized structures of **8a** (trans isomer).

3. Cyclic voltammograms of positive electrodes.

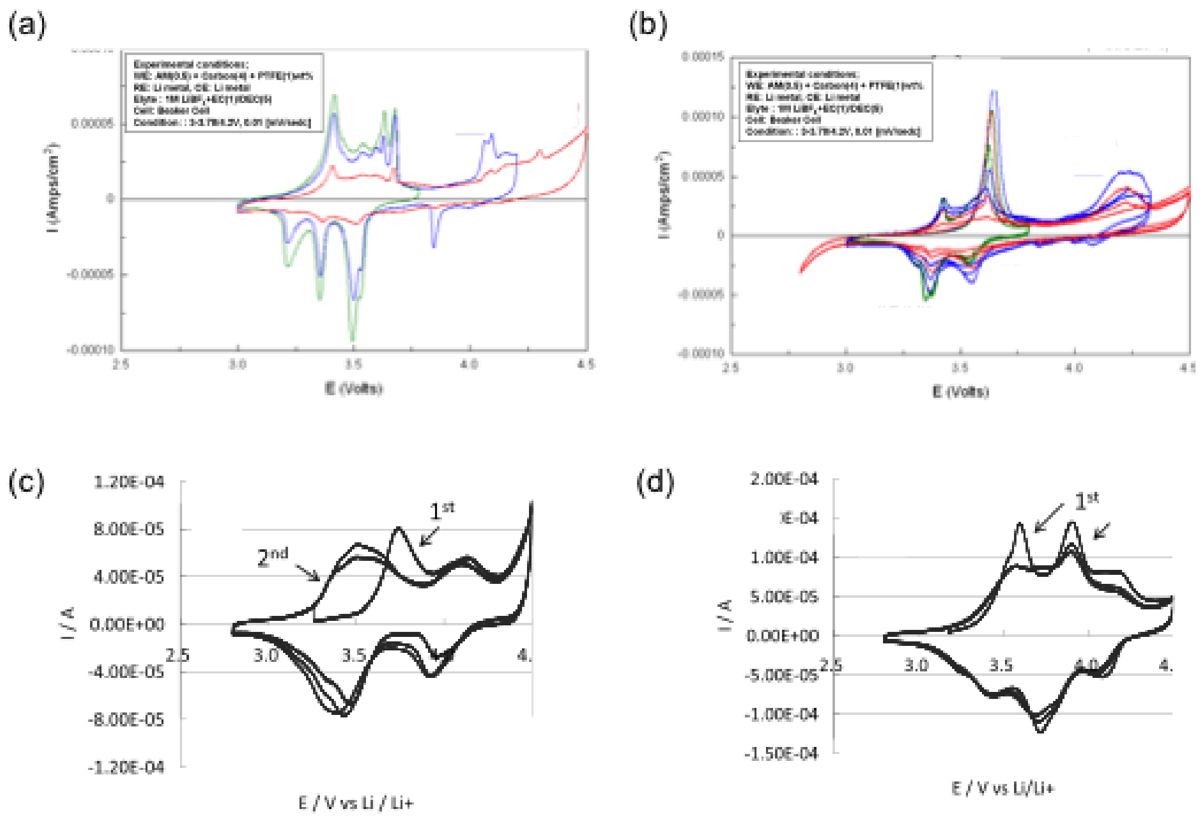

Figure S4. Cyclic voltammograms of positive electrodes using (a) **5b**, (b) **5c**, (c) **6b** and (d) **8c** as positive electrode materials.

4. Cycle-life performances for rechargeable batteries using 5b, 5c and 6b.

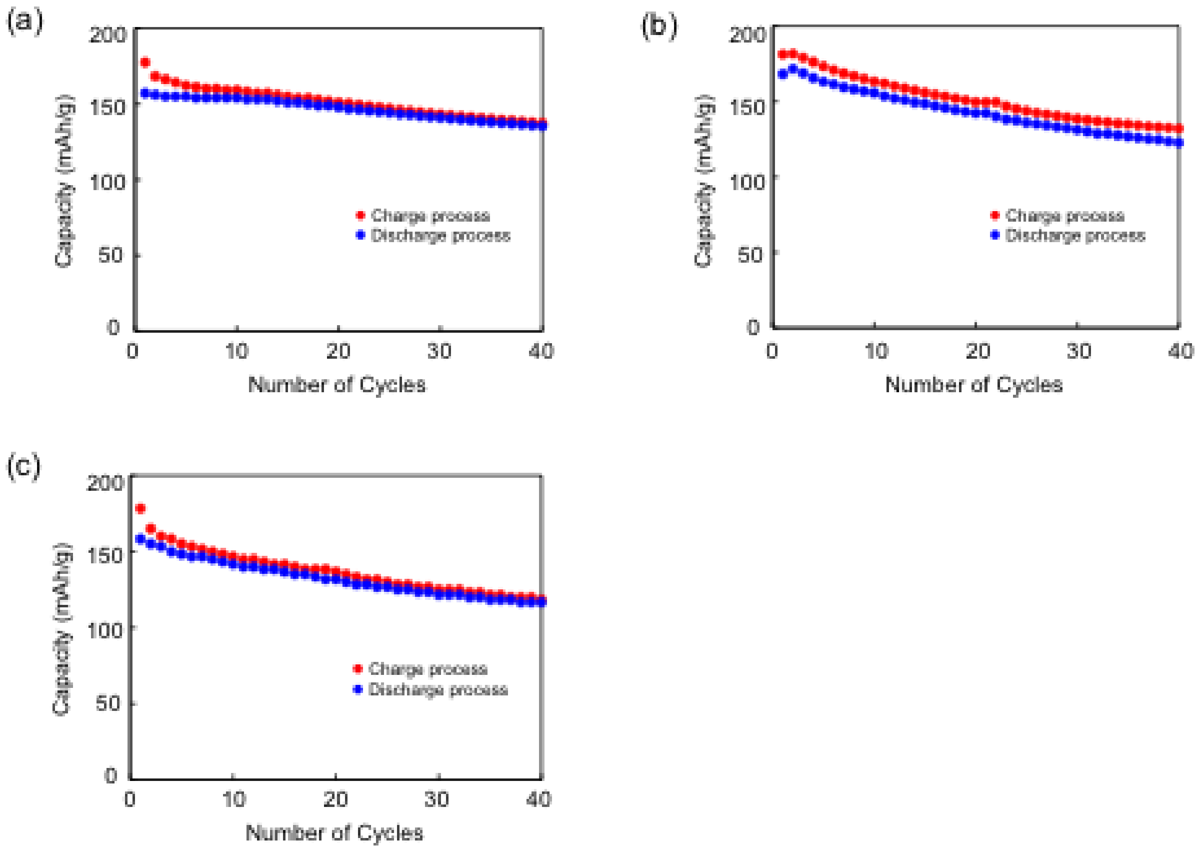

Figure S5. Cycle-life performances for (a) **5b**/Li, (b) **5c**/Li and (c) **6b**/Li cells.
